# Molecular Characterization of Tissue-Specific Anthocyanin Biosynthesis in Potato Stamens

**DOI:** 10.3390/plants14213260

**Published:** 2025-10-24

**Authors:** Sunjin Li, Zongming Guo, Xing Zhang, Huachun Guo

**Affiliations:** 1 College of Agronomy and Biotechnology, Yunnan Agricultural University, Kunming 650201, China; ynaulsj@126.com (S.L.); bryan960614@163.com (X.Z.); 2 Yunnan Engineering Research Center of Tuber and Root Crop Bio-Breeding and Healthy Seed Propagation, Kunming 650201, China; gzm_1813976450@163.com

**Keywords:** *Solanum tuberosum* L., stamen, anthocyanin biosynthesis, tissue-specific regulation, transcriptional regulation, anthocyanin composition

## Abstract

While stamen-focused research has predominantly examined flowering ornamental species, the tissue-specific regulatory mechanisms governing anthocyanin biosynthesis in potato stamens remain poorly understood. To characterize the tissue-specific regulatory mechanisms controlling anthocyanin biosynthesis in potato reproductive and storage organs, this investigation employed the red stamen mutant line ‘BF1811-8’ and the commercial cultivar ‘Atlantic’ as experimental models. Anthocyanin composition and quantification were determined using high-performance liquid chromatography (HPLC), while RNA-sequencing coupled with quantitative real-time PCR validation enabled comprehensive analysis of differential gene expression patterns within the anthocyanin biosynthetic pathway. Biochemical analysis revealed complete absence of anthocyanins across all examined tissues in ‘Atlantic’, whereas ‘BF1811-8’ exhibited tissue-specific anthocyanin profiles: stamens accumulated delphinidin and pelargonidin, while tuber skin and flesh primarily contained pelargonidin and peonidin. Gene ontology and KEGG pathway enrichment analysis of differentially expressed genes identified significant representation within secondary metabolism, flavonoid biosynthesis, and pigmentation processes. The transcription factors *StMYB4* and *StMYBA1* demonstrated positive regulatory roles in anthocyanin biosynthesis within tuber flesh and skin, respectively, while exhibiting coordinated expression with structural genes including *CHS*, *DFR*, *ANS*, and *GST*. Notably, *StbHLH94* showed stamen-specific regulatory activity and demonstrated transcriptional co-regulation with *3GT*. These findings provide crucial insights into the tissue-specific regulatory architecture governing potato anthocyanin biosynthesis, establishing a foundation for elucidating molecular mechanisms underlying tissue-specific pigmentation and advancing functional cultivar development.

## 1. Introduction

Potato (*Solanum tuberosum* L.) represents the world’s third most important food crop [1]. Beyond its primary function as a staple food source, the growing emphasis on sexual reproduction strategies in potato breeding has directed increased scientific attention toward floral organ development and function [2]. Pigmentation patterns in stamens and associated floral structures serve as critical visual cues for pollinator recognition, enhancing pollination efficiency and subsequent fruit set rates [3], while specialized coloration traits in reproductive organs contribute significantly to the ornamental value of cultivated varieties [4]. Consequently, anthocyanin accumulation in stamen tissues of mature potato flowers serves dual functions: facilitating enhanced reproductive success through improved pollinator attraction while simultaneously conferring distinctive ornamental characteristics.

Anthocyanins represent glycosylated derivatives of anthocyanin aglycones linked through glycosidic bonds. Over 1000 anthocyanidin structures occur naturally [5], with six predominant anthocyanidin aglycones characterized in plant systems: pelargonidin, cyanidin, peonidin, delphinidin, petunidin, and malvidin [6]. Previous investigations demonstrate that purple pigmentation in potato tissues predominantly comprises petunidin, peonidin, and malvidin derivatives, whereas red coloration results primarily from pelargonidin-based compounds [7]. Anthocyanin biosynthesis in pigmented potato cultivars encompasses three interconnected metabolic networks: the phenylpropanoid, flavonoid, and anthocyanin pathways, each requiring specific enzymatic catalysis [8]. The biosynthetic cascade initiates within the phenylpropanoid pathway, where phenylalanine undergoes sequential enzymatic conversion to ρ-coumaroyl-CoA via phenylalanine ammonia-lyase (PAL), cinnamic acid 4-hydroxylase (C4H), and 4-coumarate-CoA ligase (4CL). Subsequently, the flavonoid pathway converts ρ-coumaroyl-CoA to leucoanthocyanidins through the coordinated action of chalcone synthase (CHS), chalcone isomerase (CHI), flavonoid 3-hydroxylase (F3H), and dihydroflavonol reductase (DFR). The pathway culminates when anthocyanidin synthase (ANS) catalyzes the oxidation of leucoanthocyanidins to generate the corresponding colored anthocyanidins [9].

Anthocyanin accumulation in potato stamens and tubers involves complex regulatory networks governed by multiple factors, including structural genes within biosynthetic pathways and diverse transcription factors [10,11]. These regulatory mechanisms exhibit substantial tissue-specific variation in both biosynthetic capacity and transcriptional control. Structural genes encode enzymes catalyzing anthocyanin and flavonoid biosynthesis, whereas transcription factors modulate the spatiotemporal expression patterns of these structural genes [12,13]. Key transcription factor families, including MYB [14], bHLH [15], WD40 [16], and WRKY [17], regulate anthocyanin biosynthesis either independently or synergistically, resulting in distinct tissue-specific accumulation and expression profiles. Current research in rice, wheat, and maize demonstrates predominant anthocyanin accumulation within seed coat tissues [18]. Investigations demonstrate that the maize transcription factors *ZmC1* (MYB-type) and *ZmR* (bHLH family) enhance structural gene expression within the anthocyanin biosynthetic pathway, establishing tissue-specific regulatory control over pigment accumulation [19]. Furthermore, anthocyanin biosynthesis in cereal seed coats requires activation by ternary MYB-bHLH-WD40 transcriptional complexes, which coordinate expression of key biosynthetic genes including *CHS*, *CHI*, *F3’H*, and *F3H* [20,21]. Within Solanaceae species, *SmbHLH13* appears to positively regulate epidermal anthocyanin accumulation in eggplant through transcriptional activation of *SmCHS* and *SmF3H* [22]. These findings establish a comprehensive foundation for understanding tissue-specific anthocyanin biosynthetic regulation across diverse plant families.

Investigations of anthocyanin biosynthesis in potato tubers demonstrate that key structural genes within the biosynthetic pathway (*CHS*, *F3H*, *DFR*, *GST*, *F3′5′H* and *ANS*) function as positive regulators of pigment accumulation in colored cultivars [23]. Concurrently, transcriptional regulators including *StAN1*, *StAN2*, *StMYBA1*, *StMYB113*, *StbHLH1*, and *StJAF13* modulate structural gene expression through both independent and cooperative mechanisms, orchestrating anthocyanin biosynthesis across tuber and foliar tissues [24,25,26]. The transcription factor *StWRKY13* enhances promoter activity of structural genes (*StCHS*, *StF3H*, *StDFR*, and *StANS*), thereby facilitating anthocyanin biosynthesis in tuber tissues [27]. While previous investigations of potato anthocyanin biosynthesis have concentrated primarily on tuber tissues, the regulatory mechanisms governing stamen-specific pigmentation remain poorly characterized. Contemporary stamen-focused research predominantly examines ornamental flowering species, with studies identifying flavonoid metabolites, including diverse anthocyanins, as the principal chromophoric compounds in red stamen tissues of pink canna and saffron [28,29]. In peony, overexpression of *PsMYB2* promotes anthocyanin accumulation in both stamens and petals while coordinating upregulation of multiple biosynthetic pathway genes [30].

During systematic breeding efforts, we identified a spontaneous mutant line ‘BF1811-8’ exhibiting concurrent red pigmentation in both reproductive and storage organs. To characterize the tissue-specific regulatory networks governing stamen anthocyanin biosynthesis, we employed contrasting genotypes: ‘BF1811-8’ (exhibiting red pigmentation across skin, flesh, and stamens) and ‘Atlantic’ (displaying white skin and flesh with yellow stamens). We utilized HPLC (high-performance liquid chromatography) to quantify anthocyanin composition within stamen tissues, followed by RNA-sequencing analysis to identify candidate genes associated with pigment biosynthesis across reproductive and storage organs, with subsequent validation of expression patterns via qRT-PCR (quantitative real-time PCR). Comprehensive differential gene expression analysis enabled identification of key regulatory elements controlling tissue-specific anthocyanin accumulation patterns. These findings advance understanding of tissue-specific anthocyanin regulatory mechanisms in potato reproductive organs while establishing a foundation for developing cultivars with enhanced ornamental characteristics.

## 2. Methods

### 2.1. Plant Materials

Potato cultivars (lines) ‘Atlantic’ (white skin, white flesh, yellow stamens) and ‘BF1811-8’ (red skin, red flesh, red stamens) were provided by the Potato Research Institute of Yunnan Agricultural University. Among these germplasm resources, ‘BF1811-8’ constitutes a spontaneous mutant displaying anthocyanin accumulation throughout developing tissues. Pre-sprouted seed tubers were established in February using 16 cm × 12 cm cultivation containers filled with a 1:1 mixture of growing substrate and red soil, maintained under controlled greenhouse conditions. Environmental parameters included ambient lighting, constant temperature of 22 °C, and relative humidity of approximately 60%. Plants received supplemental water-soluble fertilizer applications throughout the growing period as required. During peak anthesis, tuber and stamen tissues were harvested, with potato skin (P), flesh (R), and stamens (XR) immediately flash-frozen in liquid nitrogen and preserved at −80 °C for subsequent anthocyanin quantification, transcriptome sequencing, and quantitative real-time polymerase chain reaction (qRT-PCR) analyses.

### 2.2. Anthocyanin Composition and Quantitative Analysis

Anthocyanin composition and quantification were determined using HPLC according to established protocols [31]. Standard stock solutions of six anthocyanidin reference compounds were prepared in 10% methanolic hydrochloric acid, including cyanidin (≥96%), delphinidin (≥97%), pelargonidin (≥97%), peonidin (≥97%), petunidin (≥95%), and malvidin (≥97%) (all from EXTRASYNTHESE, Genay, France). Fresh tissue samples (0.2–1.0 g) from potato skin, flesh, and stamens of ‘Atlantic’ and ‘BF1811-8’ were homogenized in acidified extraction buffer (hydrochloric acid: methanol: water, 1:2:1 *v*/*v*/*v*), mixed thoroughly, and brought to a final volume of 25 mL. Samples underwent ultrasonic extraction (XZ-10DTD Ultrasonic Cleaner, SCIENTZ, Ningbo, China) for 30 min, followed by acid hydrolysis in a boiling water bath (HH-4, JIECHENG EXPERIMENT APPARATUS, Shanghai, China) for 1 h. After cooling, samples were diluted to volume with extraction buffer and allowed to equilibrate for 8–12 h. Supernatants were collected, filtered through 0.45 μm membrane filters, and analyzed in triplicate.

Chromatographic separation was performed using an Agilent 1200 series HPLC system (Agilent Technologies, Santa Clara, CA, USA) equipped with a UV detector and ZORBAX StableBond C18 column (250 mm × 4.6 mm × 5 μm). Analytical conditions included: column temperature of 35 °C, mobile phase A consisting of 1% aqueous formic acid (>99.95%, Sigma, St. Louis, MO, USA), mobile phase B consisting of 1% formic acid in acetonitrile (>98%, Sigma, St. Louis, MO, USA), flow rate of 0.8 mL/min, injection volume of 20 μL, detection wavelength of 530 nm, and total runtime of 30 min. The gradient elution profile is detailed in Supplementary File Table S1.

### 2.3. RNA Isolation and Purification

Total RNA was extracted from stamen, tuber peel, and tuber flesh tissues using the Plant RNA Isolation Kit (Ambion, Thermo Fisher Scientific, Carlsbad, CA, USA). For each tissue type, composite samples were prepared from multiple individuals to minimize biological variation, with three independent biological replicates established per tissue type, yielding a total of 18 samples for downstream analysis.

### 2.4. cDNA Library Preparation and RNA-Sequencing Analysis

RNA samples that passed quality assessment were processed for cDNA library construction and high-throughput sequencing by Guangzhou Gene Denovo Biotechnology Co., Ltd., according to standard manufacturer protocols. Sequencing was performed using the Illumina HiSeq platform. Raw sequence data underwent comprehensive quality control procedures, including removal of adapter sequences, poly-N regions, and low-quality reads. High-quality reads were mapped against the potato reference genome DM1-3 v 6.1 (http://spuddb.uga.edu/index.shtml, accessed on 21 July 2025). Using HISAT2-Align implemented within TBtools-II software [32]. Gene expression levels were quantified using the fragments per kilobase of transcript per million mapped reads (FPKM) method [33]. Differential gene expression analysis was conducted using the DESeq2 R package 1.48.2 [34], with genes exhibiting adjusted *p*-values < 0.05 and |log2(fold change)| ≥ 1 classified as significantly differentially expressed. Functional annotation of differentially expressed genes was performed through Gene Ontology (GO) enrichment analysis and Kyoto Encyclopedia of Genes and Genomes (KEGG) pathway analysis using TB tools, identifying significantly enriched biological processes and metabolic pathways.

### 2.5. Quantitative Real-Time PCR Analysis(qRT-PCR)

Total RNA was isolated from tuber skin, flesh, and stamen tissues using the RNA simple Total RNA Kit (TIANGEN, Beijing, China). First-strand cDNA synthesis was performed using the TransScript-Uni SuperMix kit (TransGen Biotech, Beijing, China) according to manufacturer protocols, which simultaneously removes genomic DNA contamination during reverse transcription. Quantitative PCR reactions employed SYBR Premix ExTaq chemistry (TaKaRa, Tokyo, Japan) and were conducted on an Applied Biosystems 7500 Real-Time PCR System (Thermo Fisher, Carlsbad, CA, USA). Thermal cycling conditions included initial denaturation at 95 °C for 7 min, followed by 40 cycles of denaturation (95 °C, 10 s) and annealing/extension (60 °C, 30 s). Gene expression levels were normalized against the StEF1α reference gene. Specific primers were designed using SnapGene software and synthesized commercially (Sangon Biotech, Shanghai, China), with sequences detailed in Supplementary File Table S2. Relative gene expression was calculated using the 2^−ΔΔCT^ method [35].

### 2.6. Statistical Analysis and Data Visualization

Statistical analyses and data processing were conducted using Microsoft Excel 2019 and IBM SPSS Statistics 20.0. Data visualization and figure preparation employed multiple software platforms, including Microsoft Excel 2019, GraphPad Prism 8.0, TB tools, and RStudio 1.48.2. Appropriate statistical tests were applied based on data distribution and experimental design, with significance levels determined at *p* < 0.05 unless otherwise specified.

## 3. Results

### 3.1. Anthocyanin Composition and Quantitative Analysis in Potato Reproductive and Storage Tissues

**Figure 1:**
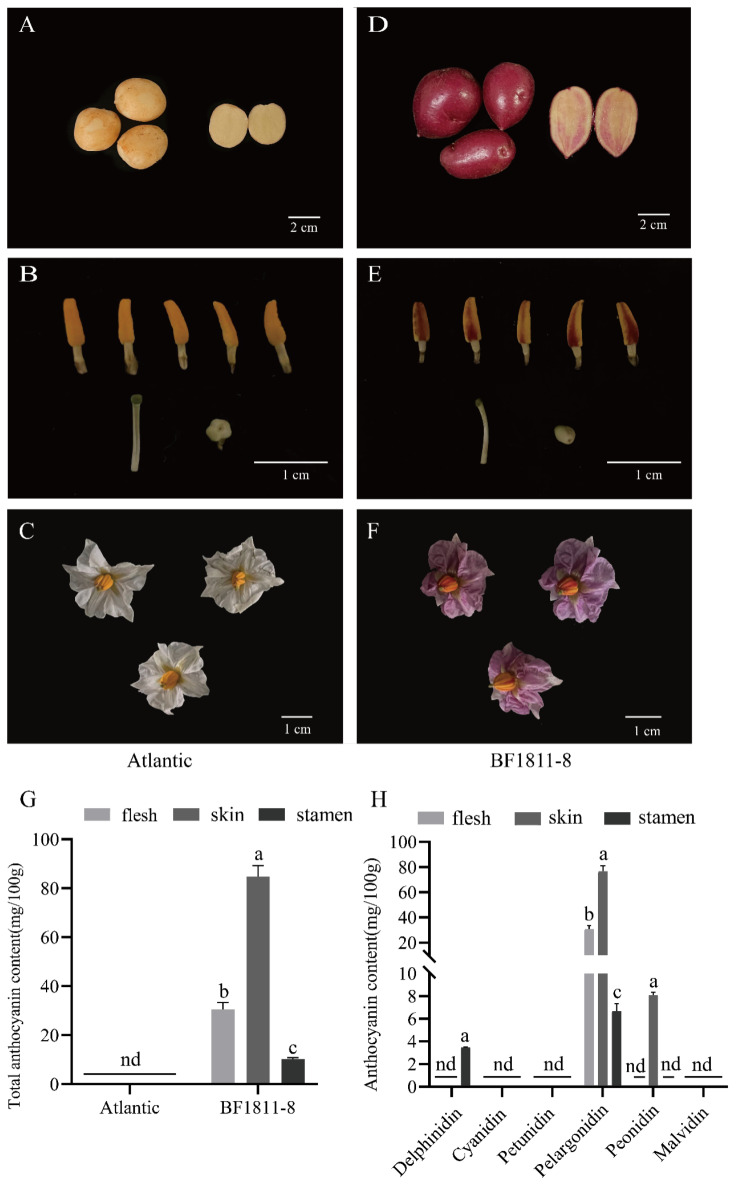
Anthocyanin component analysis in tubers and reproductive organs of potato cultivars ‘Atlantic’ and ‘BF1811-8’. (**A**–**C**). Display longitudinal section morphology, stamen phenotype, and mature flower phenotype of ‘Atlantic’, respectively; (**D**–**F**). show corresponding longitudinal section morphology, stamen phenotype, and flower phenotype of ‘BF1811-8’, respectively; (**G**). presents comparative total anthocyanin content between ‘BF1811-8’ and ‘Atlantic’ cultivars; (**H**). illustrates anthocyanin component profiles and concentrations in ‘BF1811-8’. Different lowercase letters denote statistically significant differences in anthocyanin content between cultivars across tissue types (*p* < 0.05).

Phenotypic assessment revealed distinct pigmentation patterns between genotypes (Figure 1A–F): ‘BF1811-8’ exhibited red coloration in both reproductive organs and tuber tissues, whereas ‘Atlantic’ displayed uniformly white pigmentation across corresponding structures. The pronounced red pigmentation observed in ‘BF1811-8’ stamens contrasted markedly with the pale yellow coloration of ‘Atlantic’ stamens, establishing these contrasting genotypes as suitable experimental models for investigating tissue-specific anthocyanin biosynthesis mechanisms.

HPLC analysis was conducted to elucidate the biochemical basis of observed color variations across stamen, skin, and flesh tissues in both cultivars (Figure 1G,H). Results demonstrated tissue-specific anthocyanin profiles in ‘BF1811-8’: stamens accumulated primarily pelargonidin and delphinidin, while tuber skin and flesh contained predominantly pelargonidin and peonidin. Conversely, no anthocyanin compounds were detected in any ‘Atlantic’ tissues examined.

Quantitative analysis revealed that ‘BF1811-8’ stamen tissues contained 10 mg/100 g total anthocyanins, comprising 7 mg/100 g pelargonidin and 3 mg/100 g delphinidin. Tuber skin demonstrated the highest anthocyanin concentration at 84 mg/100 g total content, with pelargonidin representing the dominant component at 77 mg/100 g. Flesh tissues exhibited intermediate accumulation levels, containing 31 mg/100 g total anthocyanins, entirely attributed to pelargonidin. In contrast, comprehensive analysis of ‘Atlantic’ tissues confirmed complete absence of detectable anthocyanin compounds across all examined organs. These biochemical findings correlated directly with observed phenotypic variations, confirming anthocyanins as the primary determinants of tissue coloration. Notably, delphinidin accumulation appeared exclusively within stamen tissues, suggesting tissue-specific biosynthetic regulation.

### 3.2. Transcriptomic Analysis of Anthocyanin Biosynthetic Gene Expression

#### 3.2.1. Differential Gene Expression Profiling

**Figure 2:**
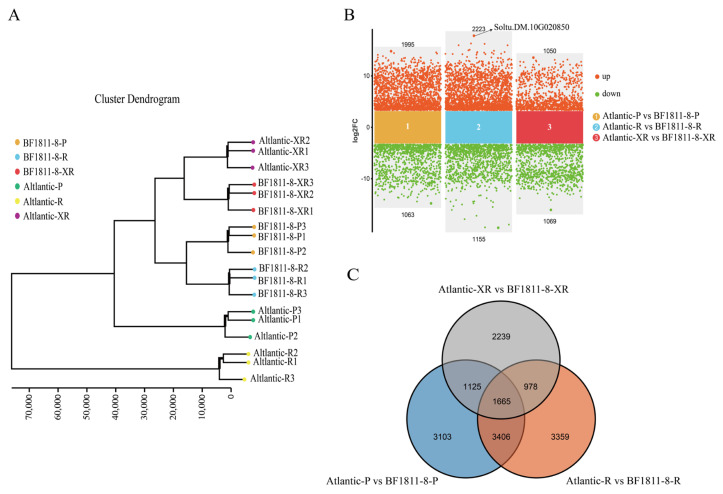
Cluster analysis and differential gene expression profiling of ‘Atlantic’ and ‘BF1811-8’ samples. (**A**) Presents hierarchical clustering analysis of sample relationships. (**B**) Displays a scatter plot visualization of differentially expressed genes. (**C**) Shows a Venn diagram illustrating overlapping differentially expressed genes across comparisons. Sample designations P, R, and XR correspond to peel, flesh, and stamen tissues, respectively.

Hierarchical clustering analysis of all samples demonstrated clear separation between experimental groups while maintaining tight clustering within biological replicates, confirming robust experimental design and sample quality for downstream analyses (Figure 2A).

Comparative transcriptomic analysis between ‘BF1811-8’ and ‘Atlantic’ revealed distinct differential expression patterns across tissue types (Figure 2B). Skin tissues showed 3058 differentially expressed genes (1995 upregulated, 1063 downregulated), flesh tissues contained 3378 differentially expressed genes (2223 upregulated, 1155 downregulated), and stamen tissues exhibited 2119 differentially expressed genes (1050 upregulated, 1069 downregulated). These results revealed tissue-specific transcriptional complexity, with flesh tissues exhibiting the highest number of differentially expressed genes, followed by skin and stamens, suggesting increasingly sophisticated regulatory mechanisms underlying anthocyanin biosynthesis in storage organs. Notably, the MYB transcription factor Soltu.DM.10G020850 exhibited the highest differential expression in skin tissues, while showing minimal expression changes in flesh and stamen tissues, indicating tissue-specific regulatory functions in anthocyanin pathway control.

Venn diagram analysis of the combined differential gene sets across all three tissue types identified 1665 commonly regulated genes between the two cultivars (Figure 2C), representing core transcriptional responses associated with anthocyanin biosynthesis regulation.

#### 3.2.2. Functional Enrichment Analysis of Differentially Expressed Genes

Functional annotation of the 1665 differentially expressed genes was conducted through Gene Ontology (GO) and Kyoto Encyclopedia of Genes and Genomes (KEGG) pathway enrichment analyses to characterize the molecular mechanisms underlying tissue-specific anthocyanin biosynthesis differences between cultivars.

**Figure 3:**
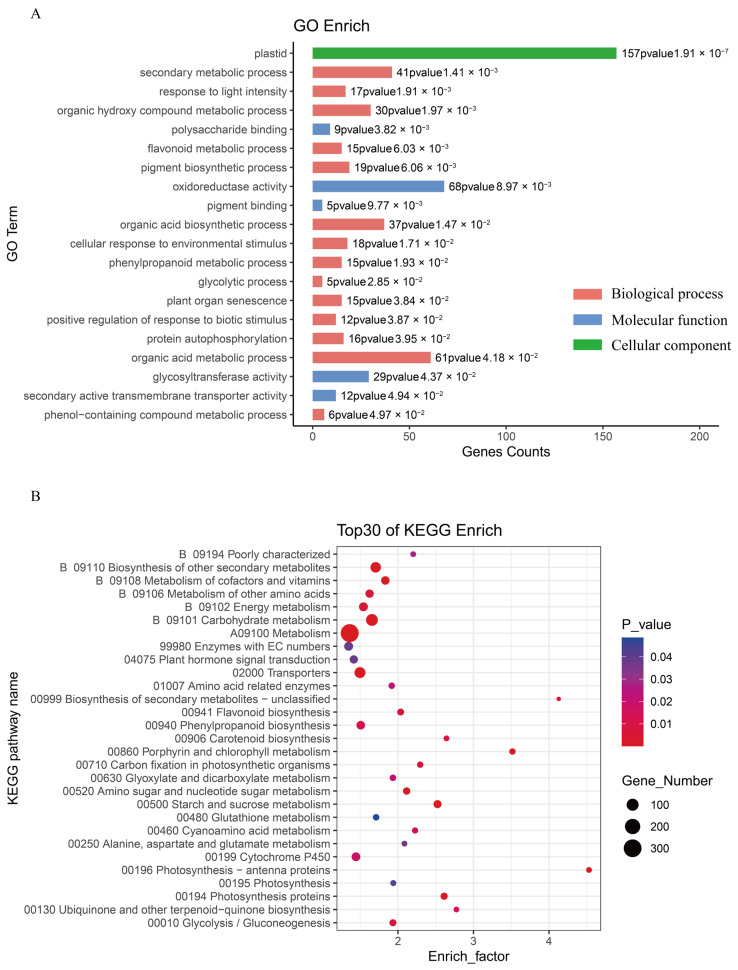
Functional enrichment analysis of differentially expressed genes between ‘Atlantic’ and ‘BF1811-8’ cultivars. (**A**) Displays Gene Ontology (GO) enrichment analysis of differentially expressed genes presented as a bar chart. (**B**) Presents KEGG pathway enrichment analysis visualized through a scatter plot format.

Gene Ontology analysis revealed significant enrichment of biological processes associated with secondary metabolism, including phenylpropanoid biosynthesis, flavonoid metabolism, organic acid processing, and pigmentation pathways (Figure 3A). Molecular function categories showed predominant enrichment in oxidoreductase activity, pigment-binding enzymes, and glycosyltransferase activity, reflecting the enzymatic requirements for anthocyanin biosynthesis and regulation.

KEGG pathway analysis identified significant enrichment in multiple interconnected metabolic networks (Figure 3B), including secondary metabolite biosynthesis, flavonoid biosynthesis, phenylpropanoid metabolism, amino acid processing, glutathione metabolism, and cytochrome-mediated processes. Additionally, pathways involved in alanine, aspartate, and glutamate metabolism showed significant representation among differentially expressed genes.

These comprehensive functional analyses demonstrated consistent enrichment of differentially expressed genes within metabolic networks directly involved in anthocyanin biosynthesis and accumulation. The identification of phenylpropanoid, flavonoid, and secondary metabolite biosynthetic pathways, along with pigmentation processes, confirms the central role of these metabolic networks in determining tissue-specific anthocyanin profiles across skin, flesh, and stamen tissues. This functional validation supports the biochemical findings and establishes a clear molecular foundation for the observed phenotypic differences between cultivars.

#### 3.2.3. Transcriptional Analysis of Anthocyanin Biosynthetic Genes

**Figure 4:**
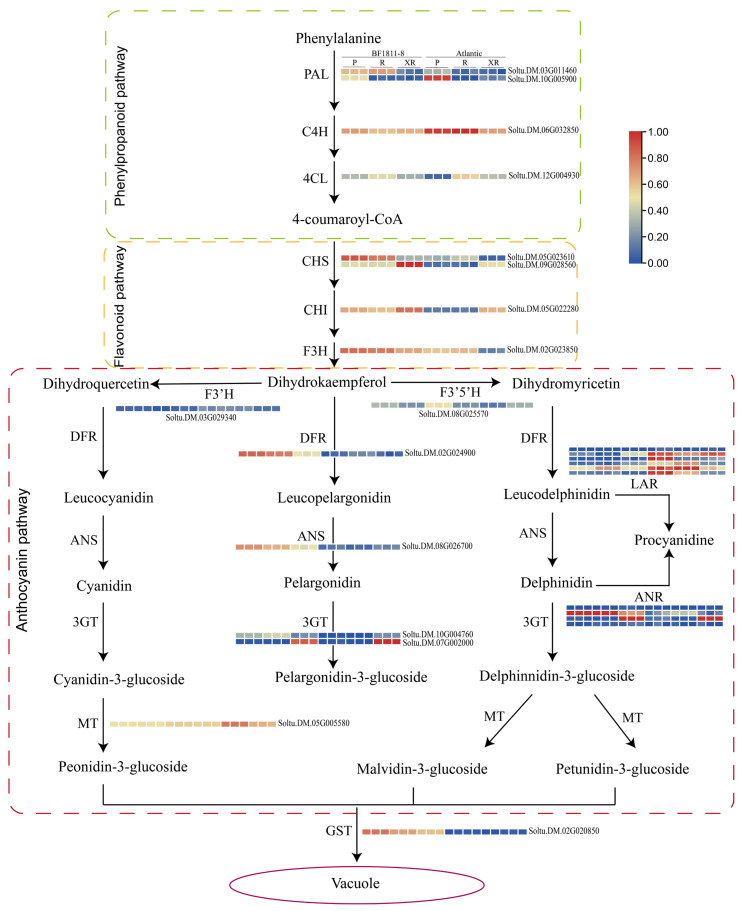
Heat map of differential structural gene expression related to anthocyanin synthesis pathway.

Expression analysis of anthocyanin biosynthetic pathway genes using transcriptomic FPKM values revealed distinct tissue-specific patterns (Figure 4). Early pathway genes *PAL1* (Soltu.DM.03G011460) and *4CL* demonstrated elevated expression in ‘BF1811-8’ tuber tissues compared to stamens, while *C4H* showed no significant differential expression between tissues.

The intermediate biosynthetic stage featured significant upregulation of *CHS* and CHI structural genes. Notably, *CHS1* (Soltu.DM.05G023610) and *CHS2* (Soltu.DM.09G028560) exhibited pronounced tissue-specific expression profiles. *CHS-1* exhibited preferential expression in tuber tissues, whereas *CHS2* showed pronounced upregulation in stamens, suggesting tissue-specific functional roles in anthocyanin biosynthesis.

Late-stage biosynthetic genes *DFR*, *ANS*, *3GT*, and *GST* demonstrated consistently higher expression in ‘BF1811-8’ compared to ‘Atlantic’ across all tissues examined. Within the proanthocyanidin biosynthetic pathway, *LAR* and *ANR* expression levels remained comparable across pigmented tissues. The pelargonidin biosynthetic enzymes *DFR*, *ANS*, and *GST* showed elevated expression in both reproductive and storage organs of ‘BF1811-8’, consistent with biochemical quantification results. *3GT2* (Soltu.DM.07G02000.1) exhibited particularly prominent expression in stamen tissues, indicating specialized regulatory functions in reproductive organ pigmentation.

These findings identify *PAL*, *CHS*, *DFR*, *ANS*, *3GT*, and *GST* as critical structural genes determining phenotypic color variation between cultivars. Specifically, *PAL1*, *CHS1*, *DFR*, *ANS*, and *GST* correlate strongly with tuber anthocyanin accumulation, while *3GT2* and *CHS2* demonstrate specialized functions in stamen-specific pigmentation processes.

#### 3.2.4. Transcriptional Regulation of Anthocyanin Biosynthesis

Transcriptional regulators serve as critical control points in anthocyanin biosynthesis pathways. Heatmap analysis of transcription factor families known to influence anthocyanin metabolism (MYB, bHLH, WRKY, bZIP) was conducted using FPKM expression values to characterize regulatory differences between cultivars and provide deeper insight into tissue-specific anthocyanin regulation mechanisms.

**Figure 5:**
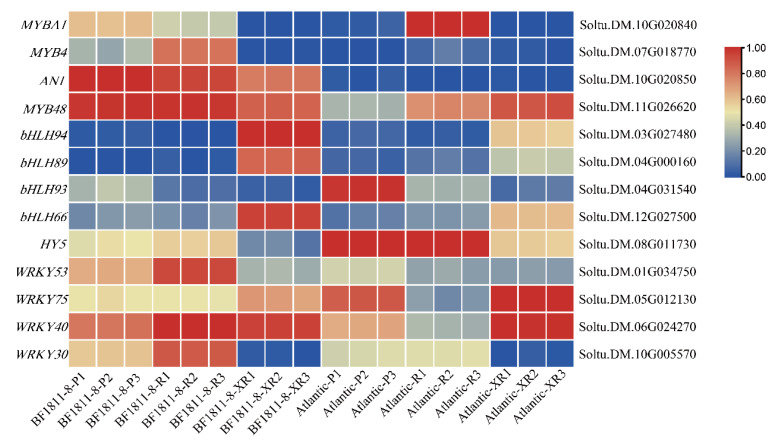
Heat map of differential transcription factor expression related to anthocyanin synthesis.

Comparative transcriptional analysis identified three transcription factors with pronounced upregulation in ‘BF1811-8’ stamen tissues: *bHLH94* (Soltu.DM.03G027480), *bHLH66* (Soltu.DM.12G027500), and *StAN1* (Soltu.DM.10G020850) (Figure 5). Among these regulators, *bHLH94* and *bHLH66* demonstrated the most substantial expression differences, suggesting specialized roles in reproductive organ pigmentation. In tuber tissues, four transcription factors showed significant upregulation: *StAN1*, *StMYBA1* (Soltu.DM.10G020840), *StMYB4* (Soltu.DM.07G018770), and *WRKY53* (Soltu.DM.01G034750). Relative to the ‘Atlantic’ cultivar, StMYB4 and StMYBA1 exhibited significantly elevated expression levels in the flesh and peel tissues of ‘BF1811-8’, respectively, suggesting these genes function as key regulatory elements in orchestrating tissue-specific anthocyanin biosynthesis.

Expression pattern analysis indicates that *StMYBA1*, *StMYB4*, and *bHLH94* function as primary regulatory determinants of tissue-specific anthocyanin biosynthesis across skin, flesh, and reproductive organs. These transcription factors represent the key molecular switches governing the observed phenotypic differences between cultivars and establish the regulatory framework for tissue-specific pigmentation control in potato.

#### 3.2.5. Regulatory Network Analysis and Gene Correlation Patterns

Correlation analysis was conducted between key structural genes and regulatory transcription factors to characterize tissue-specific anthocyanin biosynthesis patterns across reproductive and storage organs in both cultivars.

**Figure 6:**
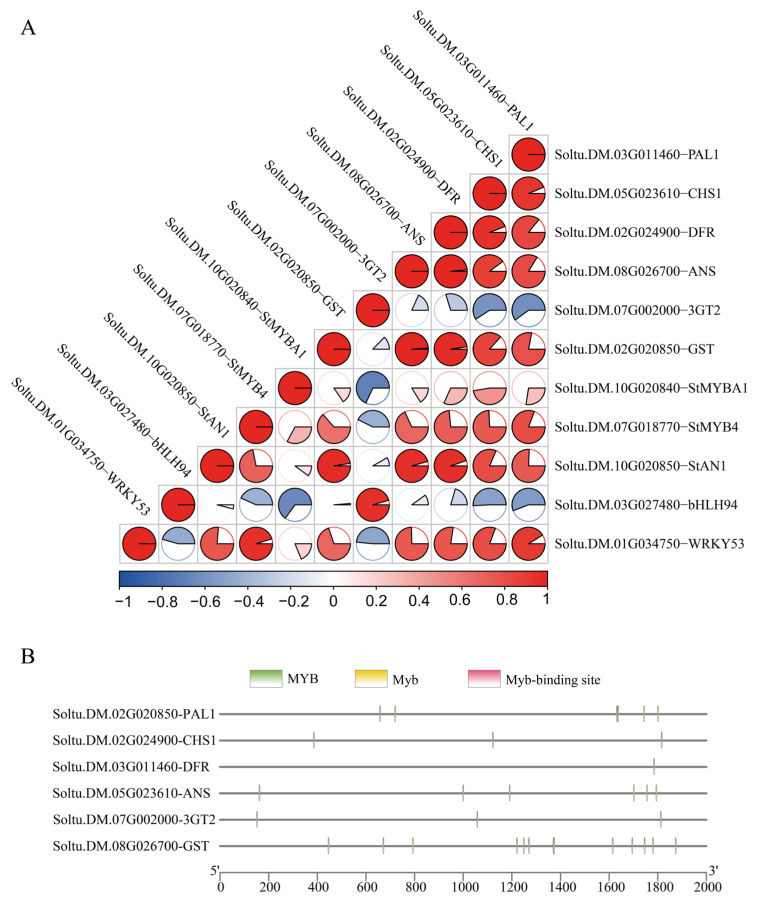
Correlation Analysis of Key Genes in Anthocyanin Biosynthesis and Promoter Cis-Acting Element Distribution. (**A**) Presents Pearson correlation coefficients among key anthocyanin biosynthesis genes (*n* = 11). (**B**) Illustrates the distribution and frequency of cis-acting regulatory elements within promoter regions.

Promoter analysis revealed that all examined structural genes contained MYB binding elements, indicating potential interactions between these transcription factors and structural genes that coordinately regulate anthocyanin biosynthesis across different tissues (Figure 6B).

The tuber-associated transcription factors *StAN1*, *StMYB4*, *StMYBA1*, and *StWRKY53* demonstrated significant positive correlations with essential biosynthetic genes (*PAL1*, *CHS1*, *DFR*, *ANS*, and *GST*), indicating coordinated regulatory networks controlling anthocyanin accumulation in storage tissues. These correlation patterns suggest that these transcription factors function cooperatively with structural genes to orchestrate tissue-specific pigmentation (Figure 6A).

The stamen-specific regulator *StbHLH94* exhibited exclusive positive correlation with *3GT2*, representing the sole transcription factor demonstrating this regulatory relationship. This unique association highlights the specialized regulatory mechanisms governing anthocyanin biosynthesis in reproductive organs and demonstrates the tissue-specific nature of transcriptional control. Analysis revealed extensive cross-correlations among transcription factor families, with *StMYB4* showing significant associations with both *StAN1* and *StWRKY53*. These inter-regulatory relationships suggest functional redundancy and cooperative regulation among different transcription factor families in coordinating anthocyanin biosynthesis across skin and flesh tissues. In contrast, *StbHLH94* exhibited significant negative correlations with *StMYB4, StMYBA1,* and *StWRKY53*, suggesting that coordinated regulation of anthocyanin biosynthesis may require the integration of these transcription factors with their respective structural gene targets. This regulatory architecture provides multiple control points for fine-tuning tissue-specific pigmentation patterns and ensures robust anthocyanin production in storage organs.

### 3.3. Quantitative Validation of Anthocyanin Biosynthetic Gene Expression

**Figure 7:**
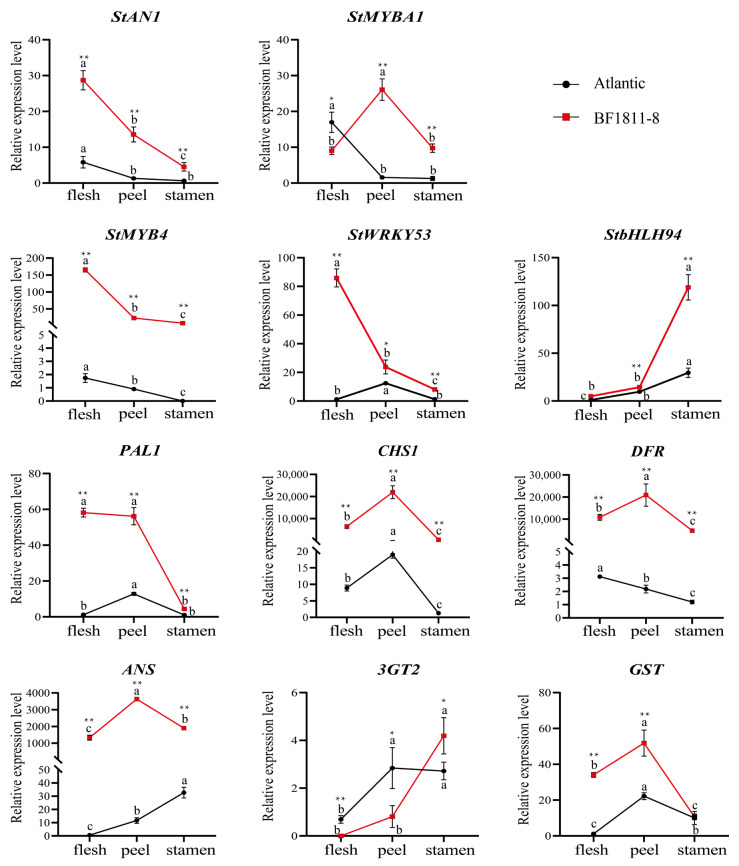
Differential expression profiles of structural genes and transcription factors involved in anthocyanin biosynthesis in tuber and stamen tissues of potato cultivars ‘Atlantic’ and ‘BF1811-8’. Statistical significance indicators: * denotes significant differences in gene expression between ‘Atlantic’ and ‘BF1811-8’ within the same tissue type (*p* < 0.05); ** indicates highly significant differences between cultivars within the same tissue type (*p* < 0.01). Different lowercase letters represent significant differences in gene expression between tissue types within each cultivar (*p* < 0.05).

qRT-PCR (Quantitative real-time PCR) validation was conducted to confirm transcriptional differences in anthocyanin biosynthetic genes between cultivars. Analysis targeted key structural genes (*PAL1*, *CHS1*, *DFR*, *ANS*, *3GT2*, *GST*) and regulatory transcription factors (*StMYBA1*, *StAN1*, *StMYB4*, *StWRKY53*, *StbHLH94*) across stamen and tuber tissues (Figure 7).

Expression analysis demonstrated consistently elevated transcript levels in ‘BF1811-8’ relative to ‘Atlantic’, corroborating both biochemical anthocyanin quantification and transcriptomic findings. Among structural genes, *CHS1* and *DFR* exhibited the highest expression levels, confirming their central roles in flavonoid and anthocyanin biosynthetic stages. The genes *PAL1*, *CHS1*, *DFR*, and *GST* showed significantly higher expression in skin and flesh tissues compared to stamens, demonstrating clear tissue-specific expression patterns. Notably, *3GT2* represented the sole structural gene with preferential stamen expression, validating its specialized function in reproductive organ pigmentation.

Transcription factor analysis revealed distinct tissue-specific regulatory patterns. The regulators *StAN1*, *StMYB4*, and *StWRKY53* demonstrated peak expression in ‘BF1811-8’ flesh tissues, while *StMYBA1* showed maximum activity in skin. The stamen-specific factor *StbHLH94* exhibited pronounced upregulation exclusively in reproductive tissues, confirming specialized regulatory functions across different organs.

Although *StMYBA1* and *StAN1* exhibited similar expression profiles, *StMYBA1* maintained elevated expression in ‘Atlantic’ flesh tissues, suggesting limited specificity for pigmented phenotypes. Conversely, *StMYB4* demonstrated exclusive upregulation in ‘BF1811-8’ flesh, indicating specialized regulatory functions in storage organ pigmentation. These validation results establish tissue-specific regulatory networks controlling anthocyanin biosynthesis and confirm the molecular basis for observed phenotypic differences between cultivars.

## 4. Discussion

### 4.1. Tissue-Specific Anthocyanin Accumulation Patterns in Potato

Established research has identified pelargonidin derivatives and peonidin glycosides as the predominant anthocyanin constituents in red potato tubers [36,37]. This investigation employed two contrasting cultivars, ‘Atlantic’ (white skin, white flesh, yellow stamens) and ‘BF1811-8’ (red skin, red flesh, red stamens), to characterize anthocyanin composition and quantification across skin, flesh, and stamen tissues.

Biochemical analysis revealed pelargonidin as the dominant anthocyanin component across all examined tissues in ‘BF1811-8’, with peonidin additionally present in skin tissues, corroborating previous findings [38] (Figure 1). Skin tissues demonstrated the highest anthocyanin accumulation at 84 mg/100 g total content, with pelargonidin representing 77 mg/100 g of this concentration. Flesh tissues contained 31 mg/100 g total anthocyanins, while stamen tissues accumulated distinct anthocyanin profiles comprising delphinidin and pelargonidin at 10 mg/100 g total content. This stamen-specific composition aligns with established profiles from colored anthers in flowering species [39,40].

Quantitative analysis demonstrated significantly lower anthocyanin concentrations in stamen tissues compared to storage organs, with the distinctive presence of delphinidin representing a tissue-specific biosynthetic signature. These compositional differences establish anthocyanin profile variation as the primary determinant of tissue-specific pigmentation patterns in potato. The exclusive occurrence of delphinidin in reproductive tissues indicates specialized metabolic pathways governing stamen pigmentation, distinguishing reproductive organ anthocyanin biosynthesis from storage tissue accumulation patterns.

### 4.2. Transcriptional Level Differences and Expression Analysis of Anthocyanin Synthesis-Related Genes in Potato Stamens and Tuber Tissues

#### 4.2.1. GO and KEGG Enrichment Analysis of Anthocyanin Synthesis-Related Genes

Previous research has established that the structural genes *CHS* and *F3’5’H* represent critical components in potato anthocyanin biosynthesis, serving as key regulatory elements that facilitate entry into the flavonoid biosynthesis pathway and subsequently promote anthocyanin accumulation [41]. Through transcriptome sequencing, we conducted GO and KEGG enrichment analyses on differentially expressed genes (DEGs) (Figure 3). The functional enrichment analysis revealed that DEGs were predominantly associated with secondary metabolic processes, phenylpropanoid metabolism, flavonoid biosynthesis, and pigment metabolism—all pathways intimately linked to anthocyanin synthesis. These findings demonstrate that flavonoid and pigment biosynthetic pathways constitute essential biological processes underlying anthocyanin production in potato stamen and tuber tissues.

#### 4.2.2. Transcriptional Level Differences and Expression Analysis of Anthocyanin Synthesis-Related Structural Genes

Transcriptional analysis of structural genes involved in the anthocyanin synthesis pathway (Figure 4) demonstrated that *PAL1*, *CHS1*, *DFR*, *ANS*, *3GT2*, and *GST* constitute key functional genes in anthocyanin biosynthesis, consistent with previous findings [42,43]. These genes represent critical structural components distributed across the phenylpropanoid pathway, flavonoid pathway, and anthocyanin synthesis pathway, respectively.

Quantitative PCR analysis of structural genes exhibiting significant transcriptional differences (Figure 7) revealed that *CHS1*, *DFR*, and *ANS* displayed markedly elevated expression levels in pigmented tissues of colored potato ‘BF1811-8’. Furthermore, expression of these genes in potato skin significantly exceeded that observed in flesh and stamen tissues, indicating that *CHS1*, *DFR*, and *ANS* serve as critical regulatory elements controlling anthocyanin synthesis in colored potato tuber tissues.

Previous studies have identified the glutathione S-transferase gene (*GST*) as a crucial component facilitating anthocyanin accumulation in the cytoplasm through vacuolar sequestration mechanisms [44]. Our analysis revealed that *PAL1* and *GST* expression in potato skin and flesh significantly exceeded levels observed in stamens, suggesting that *GST* exhibits tissue-specific regulatory functions in anthocyanin accumulation within vacuolated plant tissues.

The flavonoid 3-*O*-glycosyltransferase gene (*3GT*) represents a pivotal structural gene mediating anthocyanin synthesis in floral organs, catalyzing glycoside formation to provide essential substrates for anthocyanin biosynthesis [45,46]. Our investigation revealed elevated *3GT2* expression in stamen tissues of ‘BF1811-8’, confirming that *3GT2* exhibits specialized regulatory control in potato stamen tissues.

#### 4.2.3. Transcriptional Level Differences and Expression Analysis of Anthocyanin Synthesis-Related Transcription Factors

Recent studies have identified *StMYB180* as a candidate regulatory gene governing tissue-specific anthocyanin biosynthesis in potato corolla and abaxial leaf regions [47], demonstrating that transcription factors serve as primary regulatory elements controlling tissue-specific expression in plant anthocyanin synthesis. Transcriptome sequencing analysis revealed that among the selected MYB family genes, *StMYBA1*, *StAN1*, and *StMYB4* exhibited elevated transcriptional levels in ‘BF1811-8’ tubers (Figure 5). Notably, within the bHLH family, *StbHLH94* demonstrated high expression in ‘BF1811-8’ stamens while remaining virtually undetected in tuber tissues, indicating pronounced tissue-specific expression in stamen tissues.

*StMYBA1* expression in ‘BF1811-8’ potato skin significantly exceeded levels observed in flesh and stamen tissues, while expression in flesh remained lower than that in ‘Atlantic’, indicating that *StMYBA1* plays a prominent regulatory role in anthocyanin synthesis within skin tissues. Conversely, *StMYB4* exhibited significant expression exclusively in ‘BF1811-8’ flesh tissues. Previous research has demonstrated that *AtMYB4* in Arabidopsis exerts dual regulatory effects on flavonoid biosynthesis pathways, mediating both upstream phenylpropanoid and downstream anthocyanin biosynthesis pathways [48]. Therefore, we hypothesize that *StMYB4* may be closely associated with anthocyanin synthesis in potato flesh. Quantitative RT-PCR validation confirmed that *StMYBA1*, *StAN1*, *StMYB4*, and *StbHLH94* expression patterns aligned consistently with transcriptional levels, displaying identical tissue-specific expression trends (Figure 7).

Integrating transcriptional analysis and qRT-PCR validation results, our findings indicate that *StMYB4* and *StAN1* promote anthocyanin synthesis in potato flesh, *StMYBA1* facilitates anthocyanin synthesis in skin tissues, and *StbHLH94* enhances anthocyanin synthesis in stamen tissues. These results demonstrate that transcription factors from different families exhibit substantial expression variations across tissues, potentially reflecting distinct expression patterns or tissue-specific regulatory mechanisms. Future investigations should focus on functional validation and detailed mechanistic analysis of these genes.

### 4.3. Correlation Analysis of Tissue-Specific Expression of Anthocyanin Synthesis-Related Genes in Potato Stamen and Tuber Tissues

Recent research has elucidated the mechanisms underlying tissue-specific anthocyanin biosynthesis in various crop species. In maize, silk coloration is determined by coordinated regulation of anthocyanin biosynthesis through members of the MYB and bHLH transcription factor families [49]. The bHLH transcription factor *ZmR* specifically regulates anthocyanin biosynthesis in wheat spikes and developing seeds. Co-expression with the MYB transcription factor *ZmC1* enhances pigment accumulation across all tissue types [19]. In rice, seed coat pigmentation in purple and red varieties is mediated by MYB family members *OsMYB55* [50] and *OsMYB3* [51], along with the WD40 protein *OsTTG1* [52], which collectively regulate structural genes encoding *PAL*, *C4H*, *4CL*, *F3H*, *DFR*, and *ANS* enzymes in the anthocyanin biosynthetic pathway. Potato tuber anthocyanin biosynthesis has been investigated extensively, revealing distinct regulatory networks for different tissue compartments. The tandem R2R3-MYB genes *StMYB200* and *StMYB210* promote anthocyanin accumulation in tuber flesh [53], whereas *StMYB113* drives pigmentation in tuber periderm through direct transcriptional activation of *StF3H* and *StDFR* [25]. Additionally, the WRKY transcription factor *StWRKY70* contributes to anthocyanin biosynthesis in flesh tissues [54]. In contrast to the substantial body of research on tuber pigmentation, anthocyanin regulation in potato floral tissues remains relatively unexplored. Notably, *StFlAN2*, an AN2 ortholog within the MYB transcription factor family, has been identified as a key regulator of corolla pigmentation [55].

We conducted correlation analysis to examine the relationships between tissue-specific genes and their corresponding structural genes in anthocyanin biosynthetic pathways. Our analysis (Figure 6) revealed that the tuber-specific structural genes *PAL*, *CHS, DFR, ANS*, and *GST* exhibited highly significant correlations with the transcription factors StAN1, StMYB4, StWRKY53, and StMYBA1. These findings corroborate previous research. Specifically, *StAN1* and *StMYBA1* are homologs of *StMYB210* and *StMYB200*, respectively, demonstrating coordinated expression patterns between these transcription factors and structural genes that regulate anthocyanin biosynthesis in both skin and flesh tissues. Notably, *bHLH94*, a stamen-specific transcription factor, was the sole gene displaying positive correlation with *3GT*. Structural gene profiling demonstrated that *3GT* exhibits significant expression in the red stamens of ‘BF1811-8’, where it mediates anthocyanin biosynthesis in floral organs. Furthermore, *3GT* contains a binding motif for *bHLH94*. These observations suggest a close functional relationship between *bHLH94* and *3GT* in stamen-specific anthocyanin biosynthesis. Consequently, we propose *bHLH94* as a candidate regulatory gene for anthocyanin synthesis in stamen tissues. Additionally, *StAN1* exhibited high expression levels in both pigmented tubers and stamen tissues. Previous research confirmed its function in promoting anthocyanin synthesis in tubers and leaves [56]. This observation is consistent with prior research demonstrating that *AN2* homologs regulate anthocyanin synthesis in potato corollas, further supporting the integral role of StAN1 in stamen anthocyanin biosynthesis and establishing this gene as a central regulator of anthocyanin synthesis across multiple plant tissues.

Transcription factors regulate tissue anthocyanin biosynthesis through two complementary mechanisms: direct interaction with downstream structural genes through their own expression [57], and formation of MBW complexes with transcription factors from other families possessing complementary domains, enabling coordinated cross-regulation that achieves comprehensive transcriptional control of anthocyanin synthesis across multiple transcription factor families [58]. Within the potato WRKY family, the novel transcriptional activator StWRKY70 interacts with *StAN1* to regulate pigment synthesis in tuber flesh, potentially establishing a new paradigm for WRKY-MYB-bHLH-WD40 (WRKY-MBW) complex-mediated regulation of anthocyanin biosynthesis [54]. Our analysis revealed significant correlations among three MYB transcription factors—*StAN1, StMYB4,* and *StMYBA1*—with *StAN1* and *StMYB4* displaying particularly strong correlation. Furthermore, these MYB family members exhibited positive correlations with the WRKY family transcription factor StWRKY53. These correlations suggest the existence of feedback regulatory networks that coordinately promote anthocyanin biosynthesis. Alternatively, direct interaction between *StWRKY53* and *StAN1* may regulate anthocyanin synthesis across both reproductive and vegetative organs in potato.

## 5. Conclusions

This investigation employed high-performance liquid chromatography (HPLC) to identify the anthocyanin components in stamen tissues of the red stamen mutant line ‘BF1811-8’ as delphinidin and pelargonidin, with a total anthocyanin content of 10 mg/100 g. RNA sequencing analysis coupled with quantitative RT-PCR validation was utilized to examine transcriptional differences in anthocyanin synthesis-related genes in stamen and tuber tissues. Results demonstrated that transcription factors *StMYB4*, *StMYBA1*, *StAN1* and structural genes *CHS*, *DFR*, *ANS*, and *GST* constitute key regulatory elements governing potato tuber anthocyanin synthesis. The transcription factor *StbHLH94* and structural gene *3GT* exhibited specific regulatory functions in anthocyanin accumulation within stamen tissues. Finally, correlation analysis was conducted to further examine the associations among tissue-specific expression patterns of anthocyanin synthesis-related genes in stamen and tuber tissues. These analyses revealed potential synergistic regulatory mechanisms governing stamen tissue anthocyanin synthesis.

## Data Availability

The datasets generated and analyzed during this study have been deposited in the National Center for Biotechnology Information (NCBI) BioProject repository and are publicly accessible at http://www.ncbi.nlm.nih.gov/bioproject/1313792 (Accessed on 3 September 2025) under BioProject ID PRJNA1313792.

## Supplementary Materials

The following supporting information can be downloaded at: https://www.mdpi.com/article/10.3390/plants14213260/s1, Table S1. Elution gradients; Table S2. Sequence of qRT-PCR primers.
