# Supplementary material for: Molecular Characterization of Tissue-Specific Anthocyanin Biosynthesis in Potato Stamens
Source: Plants (Basel). 2025 Oct 24;14(21):3260. doi: 10.3390/plants14213260 (PMC12609380; doi:10.3390/plants14213260)
Supplement: Supplementary file 1 [file plants-14-03260-s001.zip › plants-3916418-supplementary.pdf]

Table S1. Elution gradients.

Time (min)	Flow rate (mL/min)	Mobile phase A (%)	Mobile phase B (%)
0	0.8	92	8
2	0.8	88	12
5	0.8	82	18
10	0.8	80	20
12	0.8	75	25
15	0.8	70	30
18	0.8	55	45
20	0.8	20	80
22	0.8	92	8
30	0.8	92	8

Table S2. Sequence of qRT-PCR primers.

Gene name	Accession number	Primer sequences
StPAL1	Soltu.DM.03G011460.1	F:GGTGTTACTACTGGATTTGGTGC
		R:CCCTTGTTGCTGAATGTGGC
StCHS1	Soltu.DM.05G023610.1	F:GGTCAGCCCAAGTCCAAGATC
		R:AGTCCCACCAGCAAAGCAACC
StDFR	Soltu.DM.02G024900.2	F:AACGGTTTGCGTCACAGGAG
		R:TCCACAGCGTTAAGTTTGTATCAGC
StANS	Soltu.DM.08G026700.1	F:CAGCTTGAGTGGGAGGATTACTTC
		R:GTTGCTAGGTTCCTGATCTGCTTG
StUFGT2	Soltu.DM.07G002000.1	F:GGATGGAACTCGATTCTGGA
		R:CACCTTCAATTTGCAGACCA
StGST	Soltu.DM.02G020850.1	F:ATGGGACACAACAGTGATTTGATCG
		R:GTGCAAAGCCACCTTCATTCATC
StAN1	Soltu.DM.10G020850.1	F:GTATGGAGAAGGAAAGTGGCATC
		R:CCTCTCTTGATATGTGGCCTTAG
StMYBA1	Soltu.DM.10G020840.1	F:GTGAAGGAAAGTGGCATCTTGTTC
		R:CTACCAGCAATAAGTGACCACC
StMYB4	Soltu.DM.07G018770.2	F:CTTGCCGCGACGAAAATGG
		R:CTACAGCTCTTCCCACACCTTGC
StbHLH94	Soltu.DM.03G027480.1	F:GAGGGATATGAATTCGGAGGATG
		R:CAGGTTTACTACTTTGTTCAGTC
StWRKY35	Soltu.DM.01G034750.1	F:GGATTTAGTTGGAGAAAGTATGGC
		R:GTGCCTTCCTTTATATGTGACTTC
StEF1α	Soltu.DM.06G005620.1	F:ATTGGAAACGGATATGCTCCA
		R:TCCTTACCTGAACGCCTGTCA
